# Solitary humpback whales manufacture bubble-nets as tools to increase prey intake

**DOI:** 10.1098/rsos.240328

**Published:** 2024-08-21

**Authors:** A. Szabo, L. Bejder, H. Warick, M. van Aswegen, A. S. Friedlaender, J. Goldbogen, J. M. Kendall-Bar, E. M. Leunissen, M. Angot, W. T. Gough

**Affiliations:** ^1^ Alaska Whale Foundation, Petersburg, PO Box 1927, AK, USA; ^2^ Marine Mammal Research Program, Hawaii Institute of Marine Biology, University of Hawaii at Manoa, Kaneohe, HI 96744, USA; ^3^ Department of Bioscience, Zoophysiology, Aarhus University, Aarhus 8000, Denmark; ^4^ University of California, Santa Cruz, CA 94720, USA; ^5^ Hopkins Marine Station, Stanford University, Pacific Grove, CA 94305, USA; ^6^ Scripps Institution of Oceanography, University of California, La Jolla, CA 92037, USA; ^7^ Department of Marine Science, University of Otago, Dunedin 9054, New Zealand

**Keywords:** tool-use, energy expenditure, prey manipulation, foraging behaviour, drones, unoccupied aerial systems

## Abstract

Several animal species use tools for foraging; however, very few manufacture and/or modify those tools. Humpback whales, which manufacture bubble-net tools while foraging, are among these rare species. Using animal-borne tag and unoccupied aerial system technologies, we examine bubble-nets manufactured by solitary humpback whales (*Megaptera novaeangliae*) in Southeast Alaska while feeding on krill. We demonstrate that the nets consist of internally tangential rings and suggest that whales actively control the number of rings in a net, net size and depth and the horizontal spacing between neighbouring bubbles. We argue that whales regulate these net structural elements to increase per-lunge prey intake by, on average, sevenfold. We measured breath rate and swimming and lunge kinematics to show that the resulting increase in prey density does not increase energetic expenditure. Our results provide a novel insight into how bubble-net tools manufactured by solitary foraging humpback whales act to increase foraging efficiency.

## 1. Introduction

Tool use can be broadly defined as ‘the external employment of an unattached environmental object to alter more efficiently the form, position, or condition of another object, another organism, or the user itself when the user holds or carries the tool during or just prior to use and is responsible for the proper and effective orientation of the tool’ [1]. While this definition is widely used, some researchers have also emphasized the purposeful nature of tool use [2] and the way tools serve as extensions of the body to solve problems for which evolution has not provided a specific morphological adaptation [3] as alternative perspectives on the phenomenon. This emphasis on intent and problem-solving highlights the role that animal cognition, innovation and ingenuity play in the evolution of tool use.

Several mammalian [2,4,5], avian [6,7], fish [8] and insect [9] lineages include species that use tools; however, while taxonomically widespread, tool-using species are relatively rare. Rarer still are species that manufacture and/or modify their tools. Well-studied examples include free-ranging chimpanzees (*Pan troglodytes*) and orangutans (*Pongo abelii*), who manufacture specialized tools for extracting insects and fruits [10–13]. Similarly, New Caledonian crows (*Corvus moneduloides*) and Goffin’s cockatoos (*Cacatua goffiniana*) manufacture wooden tools for extracting vegetation and seed matter [14–16]. Manufacturing tools in this way typically involves complex sequences of behaviour, such as selecting and detaching suitable vegetation, stripping bark and adjusting the resulting tool’s length and shape [14,17,18], to impose a novel, three-dimensional form onto natural material. This sophisticated, goal-directed behaviour, together with the comparatively large and complex brains that characterize tool-manufacturing species [19,20], has led researchers to suggest that the relative rareness of tool use and manufacture is cognitively constrained in its taxonomic distribution [11,15,19,21].

Humpback whales (Megaptera novaeangliae) are known to produce complex bubble structures— ‘bubble-nets’ [22–25]. They do so by releasing air from their blowhole as they swim in a circular path below the surface. The rising bubbles form vertical curtains that appear as one or more rings from above. Aspects of bubble-nets and net-producing whales suggest that whales manufacture these nets as foraging tools [2]. For example, the use of bubble-nets has been observed repeatedly in association with foraging in allopatric humpback whale populations [23,24,26–29]. Several researchers have noted differences in the size and shape of bubble-nets produced by whales between, and notably within, the same populations [24,25,30]. Some of these differences correlate with the number of individuals participating in the use of the net and/or the different types of prey they are targeting (e.g. Pacific herring (*Clupea pallasii*), juvenile salmon (*Oncorhynchus* spp.) and krill (Order: Euphausiacea) [24,25,30]), suggesting that whales can exert control over their structure. Indeed, humpback whales’ flexible, spindle-shaped bodies, elongated pectoral flippers with large, rounded tubercles on their leading edge and out-sized tails [29,31,32] probably provide them with sufficient manoeuvrability [33] to manufacture nets that increase foraging efficiency under specific conditions.

In this study, we use observations from individually feeding humpback whales manufacturing bubble-nets in Southeast Alaska to examine how whales employ these tools to increase their prey intake and/or to decrease their energetic expenditure. To do so, we incorporate unoccupied aerial systems (UAS, or ‘drones’) coupled with photogrammetry techniques and non-invasive animal-borne tags equipped with motion sensors and video cameras to characterize the behaviour of solitary net-producing humpback whales and the nets they produce. Specifically, we describe net features, such as bubble-net size, shape and inter-bubble distance (‘mesh size’, i.e. the horizontal spacing between neighbouring bubbles), and consider how these modifiable attributes can contribute to an increase in prey intake for net-producing whales versus non-net-producing foraging whales. We also examine breath rates, lunge kinematics and dive behaviour to explore potential energetic costs associated with deploying bubble-nets. In doing so, we provide novel insights into the benefits that tool use provides foraging whales.

## 2. Methods

This study took place at the confluence of Frederick Sound and lower Stephens Passage, northern Southeast Alaska (57°24'N, 133°31'W), between 14 and 19 July 2019. During this period, we encountered a large aggregation (>70) of humpback whales engaged in solitary bubble-net feeding. We instrumented five whales in this aggregation with high-resolution on-animal inertial-sensing CATS (Customized Animal Tracking Solutions; https://cats.is/cats-cam) data-logging video tags. Concurrently, we flew UAS over both tagged and untagged whales. We used standard photo-identification techniques to identify each focal whale’s flukes [34]. We estimated the upper and lower depths of the scattering layer, which we use as a proxy for prey depth, in proximity to the bubble-netting whales using the vessel’s dual frequency (50 and 200 kHz) Garmin GPSMAP 7621XSV depth sounder.

**Figure 1.**
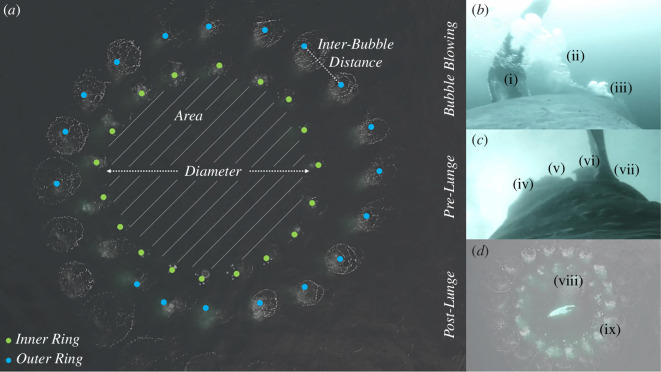
Variables and data collected from UAS and tagging methodologies. (*a*) UAS-derived bubble-net metrics for a two-ring bubble-net, including the area and diameter for the inner ring, and the horizontal inter-bubble distance (i.e. mesh size) for the outer ring. (*b*) Whale-producing bubble pulses, showing (i) left flipper, (ii) previous bubble pulse and (iii) new bubble pulse. (*c*) Whale with mouth open immediately prior to a lunge, with (iv) top jaw, (v) baleen rack, (vi) bottom jaw and (vii) right flipper visible. (*d*) Whale approaching the surface after a lunge, with the (viii) mouth closed prior to breaking the water’s surface and (ix) full bubble-net visible.

Video recordings (3840 × 2160 pixels) were obtained using a DJI Inspire 2 Pro multirotor UAS equipped with a 16 Megapixel Zenmuse X5 micro four-thirds camera with an Olympus M.Zuiko 25 mm f1.8 lens, UAS. We custom-fitted the UAS with a LightWare SF11/C laser range finder (Lightware Optoelectronics [35]) with an accuracy of 10 cm [36] to measure its altitude above sea level. We quantified whale length and fine-scale structural elements of bubble-nets, including the number of internally tangential rings within each net, the two-dimensional area (m^2^) of each ring, and the horizontal distances between neighbouring bubbles at the surface using aerial photogrammetry methods described by Christiansen *et al*. [36] (figure 1*a*).

We deployed tags on whales following previously described methods [37,38]. Whale responses to tagging were minimal, with a rapid resumption of foraging behaviour once the tagging boat had exited the animal’s immediate vicinity. The suite of sensors in each tag included tri-axial accelerometers, gyroscopes, magnetometers, a high-resolution camera, hydrophone and a pressure sensor sampled at 10 Hz, 16 bit. Using methods outlined by Cade *et al*. [39], we quantified the speed, pitch, roll and heading during net production and lunging. Using the tag-derived videos, we measured the time at which bubble-net production was initiated and the rate at which the whales released bubbles while producing the net (figure 1*b,c*). Swimming speed and depth were measured from the initiation of bubble production through the subsequent feeding lunge.

**Figure 2.**
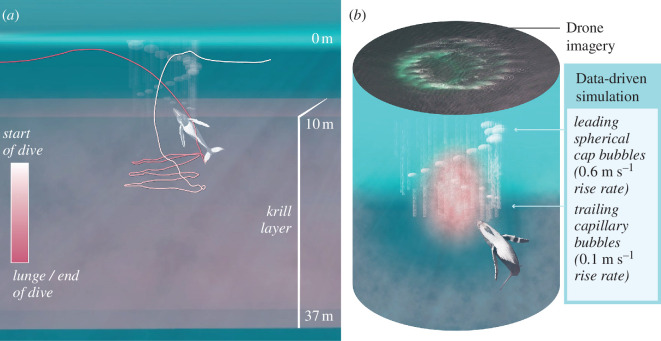
Data-driven simulation of a humpback whale manufacturing a bubble-net. (*a*) A zoomed-out view of average krill layer depth demonstrates the three-dimensional trajectory of a bubble-net feeding humpback whale from the start (white) to the end (pink) of the dive. (*b*) The UAS imagery alongside concurrent data-driven pre-lunge simulation showing 0.6 m s^−1^ rise rate of spherical cap leaders compared with 0.1 m s^−1^ rise rate for trailing capillary bubbles.

We created data-driven three-dimensional bubble-net reconstructions in Autodesk Maya using the visualization pipeline outlined in Kendall-Bar *et al*. [40], pairing sensor data for position, rotation and stroke rate to video-derived binary data for bubble pulse production. Bubble curtains were simulated using a minimum bubble rise rate of 0.1 m s^−1^ for trailing capillary bubbles and a maximum rise rate of 0.6 m s^−1^ for spherical cap leaders (figure 2). The data-driven simulation illustrates a visual hypothesis for the function of the three-dimensional bubble-net in concentrating prey within the krill layer (figure 2; electronic supplementary material, video S1).

We analysed UAS-derived aerial footage from systematic boat surveys to estimate the frequency of solitary whales manufacturing bubble-nets within the study region. These surveys were conducted between 2019 and 2021, during the first two weeks of each month from June to September throughout northern Southeast Alaska. A series of predetermined observation stations provided near-complete coverage of the study region. When whales were sighted, UAS were launched, and whales were filmed as part of a long-term whale health monitoring programme. The UAS footage was reviewed to assess the occurrence of solitary whales engaging in bubble-net foraging in the study region, as a percentage of the total number of individuals observed.

### 2.1. Statistical analyses

For our UAS dataset, we generated a series of linear mixed-effects models with the number of rings in a bubble-net as the predictor variable. The response variables for these models included the area of the innermost ring, the log_10_-transformed ratio of the innermost to the outermost ring area and the mesh sizes of the innermost and outermost rings. We also generated a linear mixed-effects model using body length as the predictor and the innermost ring area as the response variable.

For our tag dataset, we generated a linear mixed-effects model with the number of rings as the predictor and the total number of bubble pulses as the response variable. We also performed a series of paired *t*-tests (two-tailed), including a comparison between maximum dive depth and the depth at which bubble production was initiated, the number of bubble pulses produced in the innermost versus the outermost ring and the swimming speed of the whale throughout the production of the innermost versus the outermost ring.

All statistical tests were performed in RStudio (v. 1.4.1106) using the ‘stats’, ‘lme’ and ‘lmerTest’ packages [41]. We included ‘individual’ as a random effect in each linear mixed-effects model to account for between-whale variance. Significance levels were set to α = 0.05 throughout our analyses. Average values are given as mean ± s.d. throughout our study unless otherwise stated.

## 3. Results

Between 2019 and 2021, we conducted 53 systematic boat surveys during which UAS were flown. We collected 1073 high-resolution videos from 742 unique humpback whales during 301 UAS flights. Only 21 (2.8%) of these whales were engaged in solitary bubble-net foraging behaviour.

During non-systematic surveys between 14 and 19 July 2019, we photo-identified 70 individuals among a large aggregation of humpback whales engaged in solitary bubble-net feeding. The whale body length ranged from 10.4 to 13.2 m (mean ± s.d. = 11.6 ± 0.7 m), suggesting that mainly adult whales were engaged in bubble-net feeding [42]. Mean ± s.d. upper and lower depths of the shallowest prey in the vicinity of the bubble-netting whales were 10.0 ± 7.6 m and 37.2 ± 11.6 m, respectively, with an average mid-layer depth of 23.6 ± 7.5 m (*n* = 25). A second, deeper scattering layer was visible in 60% (*n* = 15) of the depth sounder images and had an average upper depth of 89.4 ± 7.3 m. In all but one image, the scattering layer was only visible at 200 kHz.

### 3.1. UAS-derived characteristics of bubble-nets

Using our UAS dataset, we documented 103 bubble-nets produced by 23 individual whales. In 20 of these bubble-nets (19.4%), a second whale was observed interacting with the focal (i.e. UAS sampled) whale and/or net. Because the nature of these interactions was unclear—e.g. if the second whale interfered with or contributed to net production—we removed those nets from our analyses. In all, 83 bubble-nets produced by solitary whales were included in this analysis; however, it was not always possible to collect all measures from all nets if, for example, we were unable to position the UAS over the net in time to capture the entire deployment or water conditions obscured certain net features. We report final sample sizes for each analysis, accordingly.

**Figure 3.**
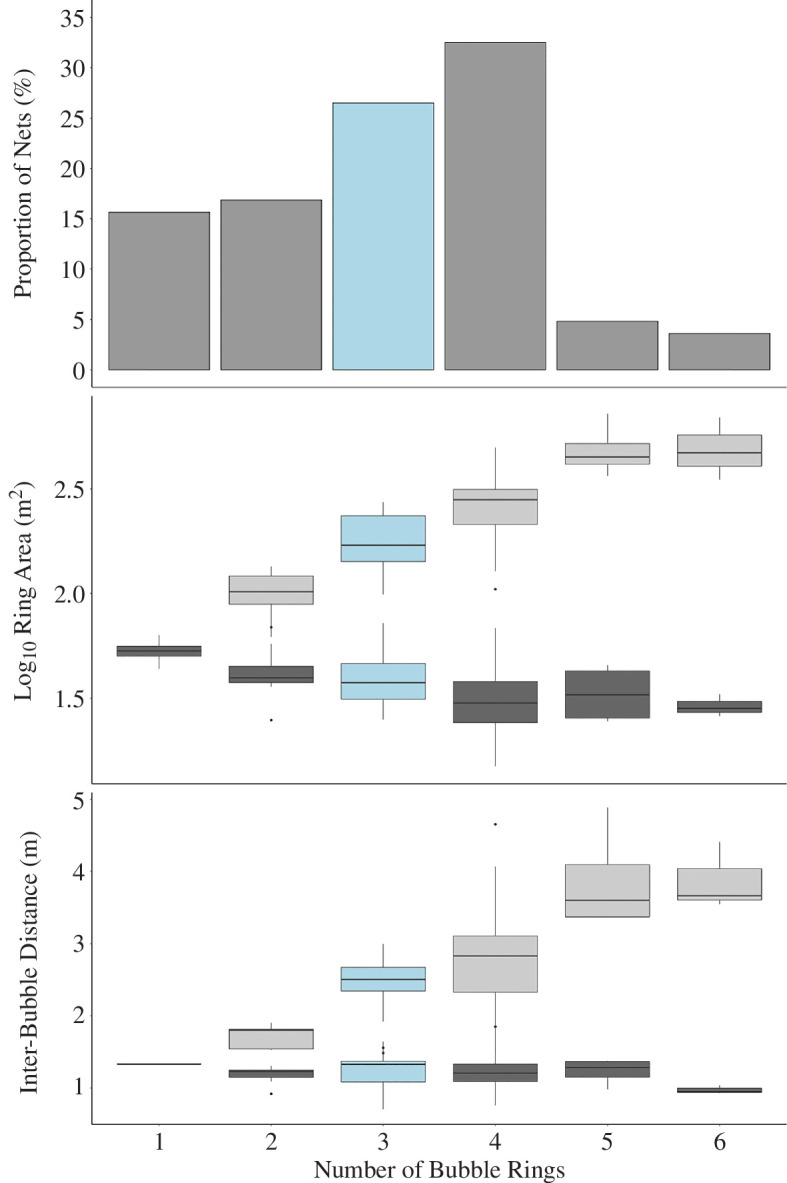
UAS-derived net measures relative to the number of rings in bubble-nets. Median ring count for all nets (3) highlighted in blue. (*a*) Proportion of nets by ring number, (*b*) log_10_ area (m^2^) of outer (light grey) and innermost (dark grey) rings. (*c*) Horizontal distance (metres) between neighbouring bubbles (mesh size) in the outer (light grey) and innermost (dark grey) rings.

Bubble-nets consisted of 1–6 (mean ± s.d. = 3.1 ± 1.3; *n* = 83) internal tangential rings (figure 3*a*). The mean ± s.d. area of the innermost ring was 37.0 ± 12.2 m^2^ (*n* = 66). The area of the innermost ring was not correlated with the whale body length (*p* = 0.216), but was inversely correlated with the number of rings in the net (*p *< 0.001; figure 3*b*). In nets with more than one ring (84.3%), the innermost ring was on average 7.2 ± 4.8 times smaller than the outermost ring (*n* = 66). There was a significant positive relationship between the log-transformed ratio of outermost to innermost ring area and the number of rings in the net (*t*_62_ = 12.3; *p *< 0.001), so adding extra rings appears to result in more concentrated prey.

The horizontal distance between bubbles in the innermost ring (1.2 ± 0.2 m; *n* = 54; figure 3*c*) was independent of the number of rings in the net (*t*_29_ = −1.05; *p* = 0.303); however, it was significantly shorter in the inner ring compared with the outermost ring (*t*_53_ = −12.228; *p *< 0.001; figure 3*c*) and the magnitude of the difference increased with the number of rings in the net (*t*_52_ = 6.4; *p *< 0.001). This suggests that the net’s ‘mesh size’ (inter-bubble distance)—i.e., the space between neighbouring bubbles through which prey could escape—is comparatively large in the outer rings and significantly smaller in the innermost ring.

### 3.2. Tag-derived data on bubble production, whale behaviour and breath rates

Using the tags, we recorded five whales producing 321 bubble-nets. In 13.7% of those bubble-nets (*n* = 44), a second whale was observed interacting with the focal whale and/or net. As with our UAS observations, data from events with multiple whales were removed from further analyses. The final dataset includes 277 bubble-net feeding events in which only the tagged whale was observed. For analyses that required precise timing of bubble initiation, we included 268 bubble-net feeding events where the initiation of bubble production could be seen or inferred clearly from the tag video.

**Figure 4.**
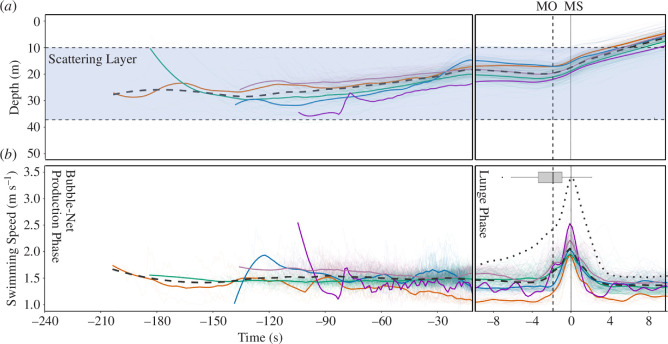
CATS tag-derived depth (*a*) and swimming speed (*b*) data relative to time (s) from five bubble-netting whales. The blue-shaded region in (*a*) represents the prey-scattering layer derived from the depth sounder images. Time = 0 corresponds to the moment when whales reach maximum speed (MS) during each event. The left and right panels loosely correspond with net-deployment and lunge phases, respectively. Change in time scale during the lunge phase (right panels) was noted to make it more easily comparable with previously published data from Gough *et al*. [43]. Line colours correspond to individual animals; faded lines represent raw data from each bubble-net deployment; bold lines represent average values for individual whales across all events; dashed lines represent average data for all whales combined and dotted line (*b*) represents the average swimming speed for humpback whales lunging without bubble-nets taken from Gough *et al*. [43]. The average scattering layer depth is shown in blue (*a*). The time when the whale opens its mouth (MO) is represented by the vertical dashed line and the associated boxplot.

Whales began deploying bubble-nets at a mean depth of 22.1 ± 4.4 m (*n* = 268; figure 4); however, the maximum depth during net deployment was, on average, 3.4 m deeper than the depth at which they initiated bubble production (paired *t*‐test, *t*_267_ = 13.852; *p *< 0.001) indicating that whales continued to descend while deploying the net. Overall, the change in depth from the whale’s maximum depth during net deployment to the depth at which it stopped producing bubbles was 7.0 ± 3.2 m (figure 4). On average, a full bubble-net took 80.0 ± 36.0 s to produce (figure 4).

While deploying nets, whales produced bubbles in discrete ‘pulses’ rather than continuous streams of air. Whales produced 7.3 (95% CI = 1.3 and 13.3) more pulses min^−1^ in the inner relative to outermost ring (paired *t*‐test; *t*_13_ = 2.6118, *p* = 0.022). The total number of individual pulses increased with the number of rings in the net (*t*_57_ = 39; *p *< 0.001).

There was no difference in whale swimming speed when deploying the inner and outermost rings (aired *t*‐test, t_204_ = 1.4872; *p* = 0.139); whales swam at an average speed of 1.5 ± 0.1 m s^−1^ throughout the deployment. However, during their final ascent through the net, whales accelerated to a maximum speed of 2.1 ± 0.2 m s^−1^ (*n* = 268; figure 4). This occurred 1.0 ± 1.8 m shallower than the depth at which they stopped producing bubbles. Cessation of bubbling occurred 10.3 ± 4.6 s prior to the whale reaching its maximal lunge speed (*n* = 268; figure 4). In the single tag deployment where the whale’s mouth was clearly visible, the whale opened its mouth during the final ascent through the bubble-net 2.2 ± 1.8 s prior to reaching its maximum lunge speed (*n* = 66 lunges; figure 4). This whale closed its mouth before reaching the surface on all dives. Mean (± s.d.) total dive cycle duration, lunge rate and breath rate were 2.86 ± 0.97 min, 24.1 ± 10.0 lunges h^−1^ and 62.9 ± 24.3 breathes h^−1^, respectively.

## 4. Discussion

Bubble-nets fit the general criteria for tool manufacture and use. Bubble-nets are unattached and employed externally by the whales. The large number of individual whales (>70) we observed using bubble-nets and the tight temporal coupling of net deployment with lunging strongly support the argument that bubble-nets confer a benefit to foraging whales. Furthermore, that there is consistency between individuals in several key, yet modifiable structural components of the nets they produce (area and inter-bubble distance in the innermost ring, and the depth of net deployment), suggests that whales exert control over the nets’ three-dimensional form to optimize that benefit. What follows is a closer examination of the net structure and the behaviour of the net-producing whales to elucidate how bubble-net tools benefit the whales and under which conditions they do so.

Several features of bubble-nets produced by solitary whales were notably consistent. All nets were produced in a clockwise direction (consistent with a right-side bias for repeated vertebrate behaviours, see MacNeilage [44]) with nearly all composed of multiple circular, internally tangential bubble rings. Each successive ring was smaller than the previous one so that the diameter of the final, innermost ring was only twice that of humpback whales’ estimated maximum gape diameter [43,45]. Also, the innermost ring’s ‘mesh size’, i.e. the horizontal spacing between neighbouring bubbles, which the whales manipulate by increasing the rate at which they produce bubbles while maintaining a constant swimming speed, was significantly smaller than that of the outer rings.

Tag video observations suggest that whales were deploying these nets to feed on euphausiids. This was supported by the fact that the scattering layer at the depth at which the whales were deploying nets was typically only visible at 200 kHz on the vessel’s echo sounder. Unlike schooling fish, such as herring, euphausiid patches, especially those that are of low density, are rarely visible at lower frequencies [46]. Thysanoessa sp. and Euphausia pacifica are common euphausiid prey for humpback whales in the region [47–51].

Field observations together with laboratory tests have shown that bubble curtains create a barrier for corralling euphausiids and schooling fish [25,30,52–55]. Whales appear to exploit the tendency for euphausiids to avoid bubbles by producing tightly nested rings that force prey initially corralled in the outermost ring into sequentially smaller rings. Given tag video limitations (i.e. insufficient video resolution and poor water clarity), quantifying krill density within the final ring would require positioning a sampling vessel equipped with a scientific echo sounder directly over a surfacing whale. This was not logistically feasible or safe for both researchers and the whales. Theoretically, however, if all prey encircled by the outermost ring are eventually concentrated into the innermost ring, this would result in, on average, a greater than sevenfold increase in prey density (i.e. proportional to the difference between the area of the outer and innermost ring). The comparatively small diameter of the innermost ring relative to the whales’ gape size ensures that much of the corralled prey can be engulfed in a single lunge. Additionally, its small mesh size probably minimizes opportunities for prey escapement when prey are most concentrated and in the nearest proximity to the surrounding bubble curtain. In this way, the various structural elements serve to both corral and concentrate prey, thereby increasing the energetic rewards gained during each lunge relative to when whales are feeding on krill in natural patch densities.

This benefit of using nets does not appear to confer an additional, direct energetic cost to the net-producing whales. Whales in our study lunged at speeds that are, on average, only 60% of those observed for whales foraging without nets (figure 4) [43]. Also, net-producing whales open their mouths prior to accelerating and actively swim through engulfment, where some portion of the mouth opening phase may occur during acceleration as predicted by Simon *et al*. [56]. This is in contrast to the typical pattern observed for lunge-feeding whales, which rapidly accelerate up to the moment they open their mouths and then coast through engulfment [43]. Swimming speed is directly correlated with energetic cost; faster swimming animals encounter higher drag and require faster and more costly tailbeats to generate the thrust required to maintain forward propulsion [57,58]. Furthermore, the average breath rates we documented for net-producing whales (62.9 ± 24.3 breathes h^−1^) were similar to the breath rates (75.1 ± 18.8 breaths h^−1^) reported for humpback whales feeding without nets when controlled for lunge rate [59]. Given the close relationship between breath rate and energy expenditure [60–62], these data suggest that, on a per-lunge basis, bubble-netting is no more energetically costly than lunge feeding without nets.

Taken together, our results suggest that, on a per-lunge basis, net-producing whales benefit from an increase in prey intake with no concomitant increase in energetic expenditure. However, despite the large number of whales we observed during our study, observations of solitary whales manufacturing bubble-nets in our study region are comparatively rare. During systematic surveys between 2019 and 2021, only 2.8% of the whales we documented were engaged in solitary bubble-net foraging behaviour. The whales we observed were not detected during surveys we conducted in the same area early in the month or at the beginning of the next month, suggesting that it was a short-duration phenomenon. Therefore, there are probably other costs associated with net production that, under most conditions, exceed the energetic benefits. Although the majority of dive cycles we observed for net-producing whales was well below the average duration for humpback whales foraging on euphausiids elsewhere [63], individuals using nets lunge only once per dive whereas whales foraging without nets can lunge as often as 15 times within a single dive [63]. Furthermore, lunge rates for humpbacks foraging without nets were as much as four times higher than we observed in our study [59]. These observations reveal that, over extended foraging bouts, net-producing whales have fewer opportunities for prey engulfment. Considering that our estimate of a greater than sevenfold increase in prey density assumes no prey escapement [64], which is unlikely, it is possible that under ‘typical’ prey conditions the actual increase in per-lunge prey intake that results from using a net is offset by the concomitant decrease in lunge rate.

Although we were unable to quantify prey density, prey patches targeted by whales in our study appeared notably more diffuse and shallower on our vessel’s depth sounder than typically observed in association with foraging whales in the region [50,51]. That we observed no whales across several days feeding without nets, which is a far more common strategy in the area, strongly suggests that under these conditions (i.e. low density and shallow prey) the use of bubble-nets by solitary individuals is favourable and, perhaps, necessary. Viewed this way, whales are not simply manufacturing tools to increase their foraging efficiency but are doing so to exploit prey aggregations that otherwise might not be energetically beneficial to forage on.

In summary, our results suggest that humpback whales manufacture bubble-net tools to increase their foraging efficiency by concentrating their prey while maintaining low energy expenditure. We further argue that this provides opportunities to exploit low-density prey patches that might otherwise not provide sufficient energetic rewards. We propose that this ingenuity has allowed humpbacks to exploit a broader ecological niche than other baleen whales, which will serve them comparatively well in the face of changing ocean conditions. However, we do not assume that nets produced by humpbacks foraging under different prey conditions or social contexts (i.e. group bubble-net feeding [22,24,25]) will be similar in structure or confer the same benefits or costs. Therefore, we recommend future studies to consider whether bubble-nets differ when produced by whales foraging under these different settings.

## Data Availability

Data are publicly available through Dryad. (doi:10.5061/dryad.m0cfxppbj) [65]. Supplementary material is available online [66].
